# The Correlation of Routine Hematological Parameters with In-hospital Mortality and Length of Hospital Stay in Patients with Large Middle Cerebral Artery Infarction

**DOI:** 10.7759/cureus.7886

**Published:** 2020-04-29

**Authors:** Muzaffer Güneş

**Affiliations:** 1 Neurology, Aksaray University Training and Research Hospital, Aksaray, TUR

**Keywords:** acute ischemic stroke, length of hospital stay, middle cerebral artery infarction, in-hospital mortality, hematological parameters

## Abstract

Objective

Neutrophil-to-lymphocyte ratio (NLR) and red blood cell distribution width (RDW) reflect inflammation and these parameters have not been studied in patients with large cerebral artery infarction. This study investigated the correlation of these inflammation parameters with in-hospital mortality and length of hospital stay (LOS) in patients with large middle cerebral artery (MCA) infarction.

Materials and methods

The study was conducted with patients who had large MCA (M1 and M2 segments) infarction. Clinical data of the patients and laboratory results at presentation were obtained from our database and recorded for statistical analysis. Multivariate logistic regression analysis was used to investigate the prognostic factors. The correlation between hematological parameters and LOS was investigated using Spearman’s Rho and Pearson correlation tests.

Results

A total of 143 patients (48 patients with hospital mortality, 95 patients without hospital mortality) were included in the study. The median LOS in patients with hospital mortality [10 (2-90) days] was significantly higher compared to the patients without [7 (2-30) days] (p = 0.024). RDW-SD was found to be a poor prognostic factor according to the multivariate logistic regression model (p = 0.004). There was a significant but weak and positive correlation between LOS and NLR (p = 0.003, r: 0.248) and a significant but weak and negative correlation between LOS and eosinophil count (p = 0.001, r: -0.278).

Conclusions

High RDW at presentation is a poor prognostic factor in terms of in-hospital mortality in patients with large MCA infarction. In addition, a positive correlation has been found between NLR and LOS.

## Introduction

There are many factors that affect in-hospital mortality in patients with acute ischemic stroke (AIS) [1]. These include the patient’s age and severity of AIS, which are the strongest determinants of prognosis in the acute phase of stroke [2]. It is also known that routine hematological parameters affect the prognosis of AIS [3]. Prolonged length of hospital stay (LOS) can result in additional complications in these patients. Previous studies have shown that LOS is associated with many factors such as hypertension, atrial fibrillation, smoking, ischemic heart disease, diabetes mellitus, severity of stroke, inpatient complications, subtype of stroke and age [4-6]. Studies have reported a relationship between neutrophil-to-lymphocyte ratio (NLR) and LOS in some diseases and medical condition, such as acute appendicitis, colorectal cancer surgery [7,8]. However, the correlation of routine hematological parameters with in-hospital mortality and LOS was not investigated in patients with large middle cerebral artery (MCA) infarction.

In patients with AIS, the prognosis can get worse as a result of complications that may develop due to prolonged LOS, which may also cause additional financial burden. There is no doubt that knowledge of the factors that affect mortality and LOS is critical. Therefore, this study was conducted to investigate the correlation of routine hematological parameters with in-hospital mortality and LOS in patients with large MCA infarction.

## Materials and methods

Subjects and study design

This study was conducted retrospectively with patients who were under follow-up and treatment for AIS in the Neurology Department of Aksaray University Training and Research Hospital between August 2014 and January 2018. Patients aged 18 years and older who were admitted to the hospital within the first 24 hours after the onset of symptoms, had M1 and M2-MCA infarction, and did not have any history of mechanical thrombectomy or intravenous thrombolytic therapy were included in the study. Patients with cerebral infarcts other than large MCA infarct (M1 and M2 occlusion), those who were not admitted to the hospital within the first 24 hours after the onset of symptoms, those under 18 years of age, pregnant women, patients who had hemorrhagic infarction, blood disorders, kidney and liver failure, malignant disease, those who received permanent immunomodulatory therapy, and those who had missing data were excluded from the study.

The patients underwent parenchymal and vascular imaging of the brain at presentation. The veins with occlusion were identified according to the cerebrovascular anatomical classification [9]. The subjects were divided into two groups, i.e. those who died of large MCA infarction in the first group and those who did not die of the same cerebrovascular occlusion in the second group (control group).

When patients are brought to the emergency department of our hospital with the preliminary diagnosis of stroke, the first step is to promptly take medical history from the patients or their relatives. The vital signs (arterial oxygen saturation, temperature, heart rate, blood pressure) are also measured rapidly and blood glucose level is measured from the fingertip. The specimens for hematology tests and biochemical analyses are obtained while conducting a swift neurological examination. Computed tomography (CT) or magnetic resonance (MR) imaging of the brain is used to obtain parenchymal images of the brain. In addition, CT angiography or MR angiography of the brain is performed for vascular imaging of the brain. The vein with occlusion is determined by parenchymal and vascular imaging of the brain and subsequently treated.

The laboratory data at the time of presentation to the emergency department was used in this study. Peripheral venous blood samples were obtained from all patients for a complete blood count and blood cell count analyses were conducted using an autoanalyzer (Sysmex XN-1000 hematology analyzer, Kobe, Japan) after centrifugation. NLR was calculated by dividing the neutrophil count by the lymphocyte count, and monocyte-to-lymphocyte ratio (MLR) by dividing the monocyte count by the lymphocyte count.

Clinical and laboratory findings at the time of presentation to the emergency department, results of the parenchymal and vascular brain imaging tests, risk factors and other demographic characteristics were obtained from our database and recorded for statistical analysis. The study was approved by the local authorities and conducted in compliance with the Declaration of Helsinki.

Statistical analysis

Results are presented as mean ± standard deviation for normally distributed data, median (min-max) for abnormally distributed data and percentage (%). To investigate the distribution pattern of the data, Kolmogorov-Smirnov normality test was used. The normally distributed variables were compared using Student’s independent samples T test, and abnormally distributed variables were compared using Mann-Whitney U test. To investigate the prognostic factors of large MCA infarction, univariate and multivariate logistic regression analysis were used. The variables with a P-value of primary comparison less than 0.25 were included in the univariate logistic regression model. Moreover, the variables with a P-value of univariate logistic regression analysis less than 0.1 were included in the multivariate logistic regression model. Hosmer-Lemeshow goodness of fit statistics were used to evaluate the model fit. Cox and Snell pseudo-R2 and Nagelkerke pseudo-R2 tests were used to assess the consistency between the variables. The correlation between two continuous variables was investigated using Spearman’s Rho correlation test and Pearson correlation test for variables without and with a normal distribution, respectively. The results were interpreted in accordance with the published Cohen’s principles (weak positive correlation, r = 0.10-0.29; moderate positive correlation, r = 0.30-0.49; strong positive correlation, r = 0.50-1.00) [10]. For statistical analysis of all data, SPSS 23.0 software was used for Windows (IBM Corp., Armonk, NY). A P-value less than 0.05 was considered statistically significant.

## Results

One hundred and forty-three patients with large MCA infarction were included in the study. The patients with hospital mortality consisted of 48 patients (19 males and 29 females, mean age: 76.3 ± 10.81 years) and the patients without hospital mortality consisted of 95 patients (47 males and 48 females, median age: 70.4 ± 11.02 years). The mortality rate of large MCA infarction was 33.6% (48 out of 143 patients). The gender distribution of the patients with and without hospital mortality was not significantly different (p = 0.263, X2 = 1.25), however, the mean age of the patients with hospital mortality was significantly greater compared to the patients without (p = 0.003).

The comparison of various blood parameters between the groups was presented in Table 1. In the Student’s T test, the mean corpuscular volume (MCV), urea and potassium did not significantly differ between the patients with and without hospital mortality (p = 0.525, p = 0.345 and p = 0.694, respectively). However, the mean red blood cell distribution width (RDW)-SD was significantly higher in patients with hospital mortality, compared to the patients without (p = 0.004). According to Mann-Whitney U test, the median C-reactive protein (CRP), white blood cell (WBC), neutrophil, monocyte, red blood cell (RBC), hemoglobin, hematocrit, RDW-CV, platelet, creatinine, sodium, alanine aminotransferase (ALT) and aspartate aminotransferase (AST) values did not significantly differ between the patients with and without hospital mortality (p = 0.697, p = 0.366, p = 0.069, p = 0.615, p = 0.469, p = 0.229, p = 0.417, p = 0.137, p = 0.434, p = 0.667, p = 0.364, p = 0.724 and p = 0.937, respectively). However, the median NLR and MLR were significantly higher (p = 0.001), and the median lymphocyte and eosinophil values were significantly lower in patients with hospital mortality (p < 0.001 and p = 0.028), compared with the patients without.

**Table 1:** The comparison of blood parameters between the groups. RDW: Red blood cell distribution width; NLR: Neutrophil / lymphocyte ratio; MLR: Monocyte / lymphocyte ratio; ALT: Alanine aminotransferase; AST: Aspartate aminotransferase; SD: Standard deviation; CV: Coefficient of variation.

	Patients without hospital mortality	Patients with hospital mortality	P-value
Age	70.44 ± 11.02	76.29 ± 10.81	0.003
RDW-SD, fL	43.64 ± 5.55	46.75 ± 6.85	0.004
Mean corpuscular volume, fL	87.17 ± 6.9	87.96 ± 7.24	0.525
Urea, mg/dL	42.28 ± 16.55	45.06 ± 17.11	0.345
Potassium, mmol/L	4.27 ± 0.48	4.3 ± 0.49	0.694
C-reactive protein, mg/dL	5.85 (0.22-79.66)	6.21 (0.54-154)	0.697
White blood cell, 10^9^/L	9.49 (5.25-19.8)	9.62 (4.7-22.93)	0.366
Neutrophil, 10^9^/L	6.23 (2.53-14.4)	6.82 (2.33-20.77)	0.069
Lymphocyte, 10^9^/L	2.22 (0.59-6.92)	1.57 (0.52-4.58)	<0.001
NLR	2.68 (0.92-21.42)	4.43 (1.18-24.36)	0.001
Monocyte, 10^9^/L	0.58 (0.12-1.49)	0.61 (0.2-1.32)	0.615
MLR	0.27 (0.4-0.88)	0.36 (0.11-1.48)	0.001
Eosinophil, 10^9^/L	0.9 (0.002-2.37)	0.055 (0.001-0.45)	0.028
Red blood cell, 10^12^/L	4.8 (3.17-7.66)	4.74 (3.3-6.03)	0.469
Hemoglobin, g/dL	13.7 (6.9-18.5)	13.65 (10.1-16.9)	0.229
Hematocrit, %	41.9 (24.2-58.2)	41.25 (29.3-50)	0.417
RDW-CV, %	13.6 (10.3-26)	14.35 (10-23.6)	0.137
Platelet, 10^9^/L	232 (135-515)	225 (102-399)	0.434
Creatinine, mg/dL	0.91 (0.42-3.4)	0.9 (0.46-1.34)	0.667
Sodium, mmol/L	140 (131-146)	139 (134-145)	0.364
ALT, U/L	14 (5-65)	15 (4-39)	0.724
AST, U/L	20 (9-52)	20 (12-43)	0.937

The Chi-square test revealed that the rates of diabetes mellitus, arterial hypertension, hyperlipidemia, congestive heart failure, coronary artery disease, valve disease and atrial fibrillation did not significantly differ between the patients with and without hospital mortality (p = 0.402, p = 0.746, p = 0.228, p = 0.058, p = 0.497, p = 0.476 and p = 0.557, respectively) (Table 2).

**Table 2:** The comparison of categorical variables (presence) between the groups.

	Patients without hospital mortality (n = 95)	Patients with hospital mortality (n = 48)	P-value	X^2^value
Gender (M/F)	47/48	19/29	0.263	1.25
Diabetes Mellitus	29 (30.5%)	18 (37.5%)	0.402	0.7
Hypertension	75 (78.9%)	39 (81.3%)	0.746	0.1
Hyperlipidemia	22 (23.2%)	7 (14.6%)	0.228	1.45
Congestive heart failure	17 (17.9%)	3 (6.25%)	0.058	3.59
Coronary artery disease	16 (16.8%)	6 (12.5%)	0.497	0.46
Valve disease	1 (1.05%)	0 (0%)	0.476	0.51
Atrial fibrillation	24 (25.3%)	10 (20.8%)	0.557	0.34

Table 3 represents the univariate and multivariate logistic regression analysis results. In the univariate logistic regression model, the age, RDW-SD, neutrophil, lymphocyte, NLR and MLR were prognostic factors (p = 0.004, p = 0.006, p = 0.012, p = 0.002, p = 0.001 and p < 0.001, respectively). However, only RDW-SD and the presence of congestive heart failure were found to be prognostic factors in the multivariate logistic regression model (p = 0.004 and p = 0.03, respectively).

**Table 3:** The univariate and multivariate logistic regression analysis results. RDW: Red blood cell distribution width; NLR: Neutrophil / lymphocyte ratio; MLR: Monocyte / lymphocyte ratio; OR: Odds ratio.

	Univariate		Multivariate	
	OR (95% CI)	P-value	OR (95% CI)	P-value
Age	1.053 (1.016-1.091)	0.004	1.033 (0.993-1.075)	0.11
RDW-SD	1.09 (1.025-1.161)	0.006	1.112 (1.035-1.196)	0.004
Neutrophil	1.155 (1.032-1.293)	0.012	1.073 (0.848-1.358)	0.558
Lymphocyte	0.513 (0.338-0.778)	0.002	0.786 (0.408-1.516)	0.473
NLR	1.199 (1.079-1.332)	0.001	1.065 (0.835-1.357)	0.614
MLR	24.34 (4.21-140.43)	<0.001	3.734 (0.372-37.436)	0.263
Congestive heart failure	3.269 (0.908-11.771)	0.07	5.769 (1.183-28.139)	0.03
Eosinophil	0.106 (0.005-2.05)	0.137	-	-
Hemoglobin	0.912 (0.764-1.088)	0.307	-	-
RDW-CV	1.052 (0.906-1.221)	0.504	-	-
Hyperlipidemia	1.765 (0.695-4.485)	0.232	-	-
			Cox and Snell pseudo-R^2 ^= 0.236	
			Nagelkerke pseudo-R^2 ^= 0.328 Hosmer-Lemeshow P = 0.173	

**Figure 1:**
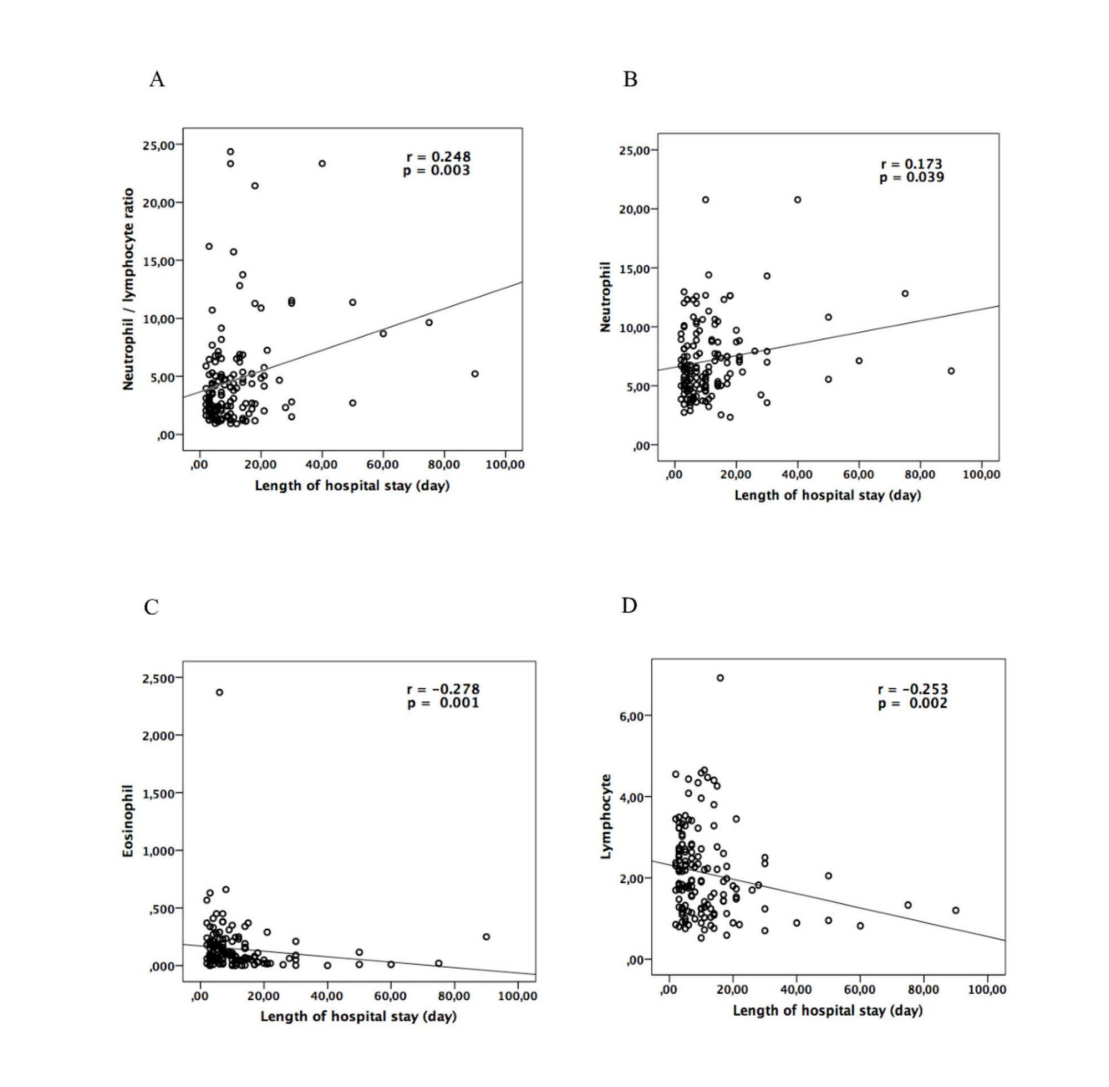
Relationship between length of hospital stay and neutrophil / lymphocyte ratio (NLR), neutrophil, eosinophil and lymphocyte.

The overall mean LOS was 11.6 ± 12.74 days (range: 2-90 days). The median LOS in patients with hospital mortality [10 (2-90) days] was significantly higher compared to the patients without [7 (2-30) days] (p = 0.024). Table 4 presents the results of the correlations between the blood test parameters and the LOS. According to the Spearman test, a significant but weak, positive correlation of the LOS with neutrophil (p = 0.039, rho: 0.173) and NLR (p = 0.003, rho: 0.248) was evident. In addition, a significant but weak negative correlation of the LOS with lymphocyte (p = 0.002, rho: -0.253), eosinophil (p = 0.001, rho: -0.278) and ALT (p = 0.037, rho: -0.175) was evident (Figure 1).

**Table 4:** Correlations between the blood test parameters and length of hospital stay. SD: Standard deviation.

	Length of hospital stay	
	Correlation coefficient (r)	P-value
Age	0.164	0.05
Red blood cell distribution width-SD	0.12	0.152
Mean corpuscular volume	0.044	0.598
C-reactive protein	0.112	0.185
White blood cell	0.108	0.201
Neutrophil	0.173	0.039
Lymphocyte	-0.253	0.002
Neutrophil / lymphocyte ratio	0.248	0.003
Monocyte	-0.065	0.442
Monocyte / lymphocyte ratio	0.134	0.11
Eosinophil	-0.278	0.001
Red blood cell	-0.03	0.722
Hemoglobin	-0.23	0.787
Hematocrit	-0.039	0.647
Red blood cell distribution width -CV	0.059	0.486
Platelet	-0.085	0.312
Creatinine	0.095	0.260
Sodium	-0.012	0.888
Alanine aminotransferase	-0.175	0.037
Aspartate aminotransferase	0.013	0.881
Urea	0.03	0.718
Potassium	0.017	0.836

## Discussion

This study has shown that high RDW is a poor prognostic factor for in-hospital mortality in patients with large MCA infarction. Moreover, it was also shown that there was a significant and positive correlation between NLR and neutrophil count at presentation and LOS, and a significant and negative correlation between eosinophil and lymphocyte counts at presentation and LOS. The study was conducted using laboratory data collected in the acute phase of the disease. All subjects were selected from patients with large MCA (M1 and M2 segments) infarction in order to obtain a relatively homogenous population.

RDW is a hematological parameter that reflects the degree of anisocytosis (variation in erythrocyte diameters) [11]. Previous studies suggested that RDW was associated with inflammatory markers such as CRP and sedimentation rate, and therefore it could be used as a parameter that indicates inflammation [12, 13]. Several recent studies reported that high RDW was associated with stroke development and with a poor prognosis in patients who had ischemic stroke [14, 15]. On the other hand, it was found in another study that RDW did not predict the severity of stroke and functional outcome in the early phase of AIS [16]. In this study, which contained a relatively more homogenous patient population than previous studies, it was found that high RDW at the time of presentation was a poor prognostic marker for in-hospital mortality in patients with large MCA infarction. It was thought that high RDW at the time of presentation could be a poor prognostic marker in patients with large MCA infarction. However, further studies are necessary.

It was reported that LOS was affected by various patient-related factors such as diabetes mellitus, age, atrial fibrillation, severity of stroke, ischemic heart disease and subtype of stroke [4, 5]. Studies investigating the correlation of LOS with hematological parameters and inflammation markers are very scarce. NLR is known as a parameter that indicates subclinical systemic inflammation [17]. According to a study published in the literature, there was a significant positive correlation between NLR and LOS [18]. Similarly, this study also demonstrated a positive correlation between neutrophil count, NLR and LOS, and a negative correlation between lymphocyte count and LOS. This study also showed a negative correlation between LOS and eosinophil count. In the light of this information, it was concluded that subclinical systemic inflammation could be a factor that prolongs LOS.

In the recent years, many studies have shown that a high NLR had a negative impact on the prognosis of AIS [19-21]. However, these studies encompassed all patients with AIS including lacunar infarction cases that can have a good prognosis and large cerebral infarction cases that can have a poor prognosis. Therefore, these studies did not have a homogenous patient population. On the other hand, this study only included patients with large MCA infarction and had a more homogenous patient population. To the best of our knowledge, there are no studies investigating NLR in patients with large MCA infarction in the literature. Unlike other studies in the literature, this study showed that NLR was a prognostic factor according to the univariate analysis, but not an independent prognostic factor according to the multivariate analysis. This was thought to stem from the homogenous patient population employed in this study. It may also stem from the fact that the study used laboratory data collected in the very early phase of stroke or that there were few patients included in the groups.

The strengths of this study were as follows: it included a relatively more homogenous patient population than the previous studies and used laboratory data that were collected at the time of presentation to the hospital. The study was retrospective, which was a limitation of this study.

## Conclusions

In conclusion, high RDW at the time of admission is useful and independent poor prognostic marker for in-hospital mortality of patients with large MCA infarction. There is a relationship between routine hematological parameters and LOS, but this relationship is weak. Even so, it is of course important to know the relationship between LOS and disease prognosis with these routine hematological parameters. In this respect, there is a need for prospective multicenter studies that include larger patient groups.
